# Supplementary material for: Passive surveillance of human African trypanosomiasis in the Democratic Republic of the Congo: clinical presentation and prospective evaluation of rapid diagnostic and reference laboratory test accuracy
Source: PLoS Negl Trop Dis. 2025 Sep 29;19(9):e0013045. doi: 10.1371/journal.pntd.0013045 (PMC12503250; doi:10.1371/journal.pntd.0013045)
Supplement: S1 File — (DOCX) [file pntd.0013045.s001.docx]

**STARD for Abstracts: essential items for reporting diagnostic accuracy studies in journal or conference abstracts**

| Section | Item |  |
| --- | --- | --- |
|  | Identification as a study of diagnostic accuracy using at least one measure of accuracy (such as sensitivity, specificity, predictive values, or AUC) | Title: Passive surveillance of human African trypanosomiasis in the Democratic Republic of the Congo: clinical presentation and prospective evaluation of rapid diagnostic and reference laboratory test accuracy. |
| **Background and Objectives** | Study objectives | Line 22-5: Passive screening of gambiense human African trypanosomiasis (HAT) is based on rapid diagnostic tests (RDT), but sensitivity of the currently commercialised RDTs has hardly been assessed prospectively. In view of the increasing importance of remote testing for HAT, the diagnostic performance of reference laboratory tests also needs further documentation. |
| **Methods** | Data collection: whether this was a prospective or retrospective study | Title: Passive surveillance of human African trypanosomiasis in the Democratic Republic of the Congo: clinical presentation and prospective evaluation of rapid diagnostic and reference laboratory test accuracy. |
|  | Eligibility criteria for participants and settings where the data were collected | Line 27-8: Clinical suspects in 29 health facilities in DR Congo were screened between October 2017 and December 2020 |
|  | Whether participants formed a consecutive, random, or convenience series | Line 28: Clinical suspects in 29 health facilities in DR Congo were screened consecutively between October 2017 and December 2020 with 3 HAT RDTs, including HAT Sero K-SeT, an RDT that is nowadays still commercialized. |
|  | Description of the index test and reference standard | Line 27-31: Clinical suspects in 29 health facilities in DR Congo were screened consecutively between October 2017 and December 2020 with 3 HAT RDTs, including HAT Sero K-SeT, an RDT that is nowadays still commercialized. HAT RDT positives were examined parasitologically and their dried blood spots tested in trypanolysis, indirect ELISA/T.b. gambiense, LAMP Trypanosoma brucei Detection Kit and m18S and TgsGp qPCR. Association of clinical signs with HAT, and sensitivity, specificity, and predictive values of the screening and reference laboratory tests were estimated using parasitology as the gold standard. |
| **Results** | Number of participants with and without the target condition included in the analysis | Line 33: Trypanosomes were detected in 42/3113 study participants. |
|  | Estimates of diagnostic accuracy and their precision (such as 95% confidence intervals) | Sensitivity and specificity of HAT Sero K-SeT were respectively 100% (42/42; 95% CI 91.6-100%) and 93.9% (2882/3071; 95% CI 92.9-94.6%). Specificities of the reference laboratory tests were ≥ 91.6%, except for LAMP. Sensitivity of ELISA/T.b. gambiense and trypanolysis were 93.9% (31/33; 95% CI 80.4-98.9) and 84.9% (28/33; 95% CI 69.1-93.4), and were ≤ 63.6% for LAMP, m18S and TgsGp qPCR. |
| **Discussion** | General interpretation of the results | Line 43-6: Compared to the WHO’s target product profiles for gambiense HAT RDTs, the HAT Sero K-SeT RDT had ideal sensitivity but its specificity was on the borderline of minimally acceptable. Sub-optimal sensitivities of trypanolysis and to a lesser extent, indirect ELISA/T.b. gambiense when applied on DBS, were confirmed. Molecular tests for remote testing need to be improved and evaluated further. |
|  | Implications for practice, including the intended use of the index test | Line 43-6 as above.  But also intro line 22-5: Passive screening of gambiense human African trypanosomiasis (HAT) is based on rapid diagnostic tests (RDT), but sensitivity of the currently commercialised RDTs has hardly been assessed prospectively. In view of the increasing importance of remote testing for HAT, the diagnostic performance of reference laboratory tests also needs further documentation. |
| **Registration** | Registration number and name of registry | Line 27: The study is registered in ClinicalTrials.Gov under identifier NCT03356665. |

*Cite this as: Cohen JF, Korevaar DA, Gatsonis CA, Glasziou PP, Hooft L, Moher D, Reitsma JB, de Vet HCW, Bossuyt PM, for the STARD Group. STARD for Abstracts: Essential items for reporting diagnostic accuracy studies in journal or conference abstracts. BMJ 2017;358:j3751*
