# Supplementary material for: Passive surveillance of human African trypanosomiasis in the Democratic Republic of the Congo: clinical presentation and prospective evaluation of rapid diagnostic and reference laboratory test accuracy
Source: PLoS Negl Trop Dis. 2025 Sep 29;19(9):e0013045. doi: 10.1371/journal.pntd.0013045 (PMC12503250; doi:10.1371/journal.pntd.0013045)
Supplement: S2 File — (DOCX) [file pntd.0013045.s002.docx]

|  | **Section & Topic** | **No** | **Item** | **Reported on page #** | **Text** |
| --- | --- | --- | --- | --- | --- |
|  |  |  |  |  |  |
|  | **TITLE OR ABSTRACT** |  |  |  |  |
|  |  | **1** | Identification as a study of diagnostic accuracy using at least one measure of accuracy  (such as sensitivity, specificity, predictive values, or AUC) | 2 | Line 1-3: Passive surveillance of human African trypanosomiasis in the Democratic Republic of the Congo: clinical presentation and prospective evaluation of rapid diagnostic and reference laboratory test accuracy.  Line 30-31: Association of clinical signs with HAT, and sensitivity, specificity, and predictive values of the screening and reference laboratory tests were estimated using parasitology as the gold standard. |
|  | **ABSTRACT** |  |  |  |  |
|  |  | **2** | Structured summary of study design, methods, results, and conclusions  (for specific guidance, see STARD for Abstracts) | 2 | Structured summary of background, Methodology/Principal Findings, Conclusions/Significance following PLOS NTD guidelines |
|  | **INTRODUCTION** |  |  |  |  |
|  |  | **3** | Scientific and clinical background, including the intended use and clinical role of the index test | 4 | Line 69-73: Positivity in serological screening is a first step towards microscopic confirmation and live saving treatment in case trypanosomes are detected [1,2]. Moreover, with the introduction of new effective and safe oral drugs [3,4], widened treatment of seropositives, without mandatory parasitological confirmation, also called a “screen and treat” strategy, is increasingly considered [5,6].  Line 84-87: In addition, the diagnostic performance, and in particular sensitivity, of remote reference laboratory tests for HAT also needs to be documented in view of their growing importance for country verification of elimination and to follow up HAT epidemiology once “screen and treat” is introduced [6,16]. |
|  |  | **4** | Study objectives and hypotheses | 5 | Line 88-95: We here report on a prospective diagnostic performance evaluation of 3 HAT RDTs, HAT Sero K-SeT, SD Bioline HAT and rHAT Sero-Strip, during passive screening in the Democratic Republic of the Congo (DR Congo). This evaluation in DR Congo, the country reporting three quarters of all gambiense HAT cases, was part of the multi-country DiTECT-HAT-WP2 diagnostic study (NCT03356665) of which the results obtained in Côte d’Ivoire and Guinea have been published previously [12,13]. Taking into account that the SD Bioline HAT and rHAT Sero-Strip RDTs used in the study are not available anymore, we will focus on diagnostic performance of clinical symptoms and signs, of HAT Sero K-SeT, and of the remote reference laboratory tests performed on dried blood spots (DBS). |
|  | **METHODS** |  |  |  |  |
|  | *Study design* | **5** | Whether data collection was planned before the index test and reference standard  were performed (prospective study) or after (retrospective study) | 6 | Line 113-115: Study participants in DR Congo were recruited prospectively and consecutively between October 2017 and December 2020 among HAT clinical suspects presenting in 29 health facilities in the provinces of Kinshasa and Kwilu (Fig 1). |
|  | *Participants* | **6** | Eligibility criteria | 7 | Line 134-139: Inclusion criteria were presence in a HAT endemic area; and presenting with clinical suspicion for HAT (recurrent fever not responding to anti-malarial medication; persistent headache; enlarged cervical lymph nodes; important weight loss; weakness; severe itching; amenorrhea, abortion, or sterility; coma; psychiatric problems; sleep perturbation; motor disorders; or speech disorders). Exclusion criteria were having been treated previously for HAT; absence of written informed consent (and assent for minors); or being less than 4 years old. |
|  |  | **7** | On what basis potentially eligible participants were identified  (such as symptoms, results from previous tests, inclusion in registry) | 7 | Line 134-139: Inclusion criteria were presence in a HAT endemic area; and presenting with clinical suspicion for HAT (recurrent fever not responding to anti-malarial medication; persistent headache; enlarged cervical lymph nodes; important weight loss; weakness; severe itching; amenorrhea, abortion, or sterility; coma; psychiatric problems; sleep perturbation; motor disorders; or speech disorders). Exclusion criteria were having been treated previously for HAT; absence of written informed consent (and assent for minors); or being less than 4 years old. |
|  |  | **8** | Where and when potentially eligible participants were identified (setting, location and dates) | 6 | Line 113-115: Study participants in DR Congo were recruited prospectively and consecutively between October 2017 and December 2020 among HAT clinical suspects presenting in 29 health facilities in the provinces of Kinshasa and Kwilu (Fig 1).  Line 119-124: The SSS in Kinshasa province were the health posts or centers of Dingi Dingi, Kikimi, Kimbuala, Liboke, Mama Marie, Menkao, Mokali, Ngamanzo and Sophora, and the hospital of Mbankana. The SSS in Kwilu were health posts or centers of Bagata 1, Ebay, Kama, Kisakinda, Lumbu, Masamuna, Mpene I, Musaba, Mushie Pentane, Saint Joseph and Saint Paul II Etna. In Kinshasa, the Maluku and Roi Baudoin hospitals acted as CDT, in Kwilu the health reference centres and hospitals of Bandundu, Bangumi, Bagata, Nkara, Masamuna and Mokala. |
|  |  | **9** | Whether participants formed a consecutive, random or convenience series | 6 | Line 113-115: Study participants in DR Congo were recruited prospectively and consecutively between October 2017 and December 2020 among HAT clinical suspects presenting in 29 health facilities in the provinces of Kinshasa and Kwilu (Fig 1). |
|  | *Test methods* | **10a** | Index test, in sufficient detail to allow replication | 7 | Line 132-4: The study protocol and tests carried out were the same as in Côte d’Ivoire and Guinea [12,13]. The study documents and all standard operating procedures of tests mentioned below are deposited in https://doi.org/10.23708/LTTOWL. |
|  |  | **10b** | Reference standard, in sufficient detail to allow replication | 7 | Line 132-4: The study protocol and tests carried out were the same as in Côte d’Ivoire and Guinea [12,13]. The study documents and all standard operating procedures of tests mentioned below are deposited in https://doi.org/10.23708/LTTOWL. |
|  |  | **11** | Rationale for choosing the reference standard (if alternatives exist) | 8 | Line 142-8: HAT RDT negative participants were considered HAT free, while participants positive in at least one RDT were considered serological suspects and referred for parasitological examination. If lymphadenopathy was present, lymph was collected and microscopically examined. If no lymphadenopathy was present or lymph was parasite negative, 4 ml of heparinized blood was examined using the mini anion exchange centrifugation technique on buffy coat (mAECT-BC)[17]. The cerebrospinal fluid (CSF) of parasitologically confirmed HAT patients and of RDT seropositives with strong clinical suspicion was examined for cytorachia and for presence of trypanosomes [18]. |
|  |  | **12a** | Definition of and rationale for test positivity cut-offs or result categories  of the index test, distinguishing pre-specified from exploratory | 7 | The study protocol and tests carried out were the same as in Côte d’Ivoire and Guinea [12,13]. The study documents and all standard operating procedures of tests mentioned below are deposited in <https://doi.org/10.23708/LTTOWL>. The cut-offs are in the SOPs. |
|  |  | **12b** | Definition of and rationale for test positivity cut-offs or result categories  of the reference standard, distinguishing pre-specified from exploratory | 9 | Line 171-3: Only participants with trypanosomes were considered HAT positive. Participants that were HAT RDT negative or in whom no trypanosomes could be detected after microscopic examination(s), were considered HAT negative. |
|  |  | **13a** | Whether clinical information and reference standard results were available  to the performers/readers of the index test | 10 | Figure 2 (flow chart) |
|  |  | **13b** | Whether clinical information and index test results were available  to the assessors of the reference standard | 10 | Figure 2 (flow chart) |
|  | *Analysis* | **14** | Methods for estimating or comparing measures of diagnostic accuracy | 9 | Line 171-3: Only participants with trypanosomes were considered HAT positive. Participants that were HAT RDT negative or in whom no trypanosomes could be detected after microscopic examination(s), were considered HAT negative. |
|  |  | **15** | How indeterminate index test or reference standard results were handled |  | Not applicable |
|  |  | **16** | How missing data on the index test and reference standard were handled | 9 | Line 167-8: Participants who were RDT positive, but did not undergo parasitological examination, or had missing or inconsistent basic data were excluded from the analysis.  Figure 2 |
|  |  | **17** | Any analyses of variability in diagnostic accuracy, distinguishing pre-specified from exploratory |  | Not applicable |
|  |  | **18** | Intended sample size and how it was determined | 7 | The study protocol and tests carried out were the same as in Côte d’Ivoire and Guinea [12,13]. The study documents and all standard operating procedures of tests mentioned below are deposited in <https://doi.org/10.23708/LTTOWL>. The sample size calculation is in the study protocol. |
|  | **RESULTS** |  |  |  |  |
|  | *Participants* | **19** | Flow of participants, using a diagram | 10 | Figure 2 (flow chart) |
|  |  | **20** | Baseline demographic and clinical characteristics of participants | 10-12 | Line 193-206 (including table 1: Study results from 3113 participants were therefore analysed (Fig 2). In the province of Kinshasa 618/3113 (19.9%) participants were recruited, in the province of Kwilu 2495/3113 (80.1%). In total 1478/3113 (47.5%) participants were included in an SSS, and 1635/3113 (52.5%) in a CDT. Most participants were female 1693/3113 (54.4%), the median age was 29 years (interquartile range: 19-43). The most frequent clinical presentations were headache (61.8%) and recurrent fever (61.2%), followed by weight loss (25.9%), and weakness (25.5%) (Table 1). Overall, 253/3113 study participants were positive in at least one HAT RDT (8.1%; 95% CI: 7.2- 9.1%), including 231/3113 in HAT Sero K-SeT (Fig 2). |
|  |  | **21a** | Distribution of severity of disease in those with the target condition | 12 | Line 214-6: The median CSF white blood cell count of the HAT patients was 244/µl (n=22/42, range: 3-2471/µl), among the 22 HAT patients who underwent staging, there was only one stage 1 patient. |
|  |  | **21b** | Distribution of alternative diagnoses in those without the target condition |  | Not applicable |
|  |  | **22** | Time interval and any clinical interventions between index test and reference standard | 12 | Line 201-214: Parasitological confirmation was immediate -upon the first parasitological examination- for 40 HAT patients, of which 7 in lymph (not tested anymore in mAECT-BC) and 26 in mAECT-BC. Examination of the CSF was parasite positive in 14 HAT patients (out of 20 with CSF examined for trypanosome presence), of which 7 had not been previously confirmed by lymph or blood examination. One HAT patient was confirmed after a second parasitological examination at the first follow up visit 7 months post-inclusion (mAECT-BC positive), and another one after a third parasitological examination at the 2nd follow-up 17 months after inclusion (mAECT-BC positive). |
|  | *Test results* | **23** | Cross tabulation of the index test results (or their distribution)  by the results of the reference standard | 10 | Figure 2: Flow chart of DiTECT-HAT-WP2 study participants. |
|  |  | **24** | Estimates of diagnostic accuracy and their precision (such as 95% confidence intervals) | 14-16 | Table 2 &3 |
|  |  | **25** | Any adverse events from performing the index test or the reference standard |  | Not applicable |
|  | **DISCUSSION** |  |  |  |  |
|  |  | **26** | Study limitations, including sources of potential bias, statistical uncertainty, and generalisability | 18-19 | Line 329- 353: This study in DR Congo had some limitations. Some of these are inherent to the DiTECT-HAT-WP2 study design and have been discussed elsewhere [12,13]: non-inclusion of individuals without the selected clinical signs and symptoms, the lack of parasitological examination of RDT negatives, imperfect gold standard, inclusion of almost exclusively 2nd stage HAT patients in passive screening, some subjectivity in assessing clinical symptoms and signs and only fragmentary follow-up for parasite negative RDT positives, even for those with positive reference laboratory tests. In the present study, in particular the retrospective exclusion of 16.4% of the study participants is an important weakness, and shows the challenges of conducting a multi-centre diagnostic trial with most health staff having little or no clinical trial experience, in hard to reach remote settings where HAT typically occurs. Of the excluded individuals, about half (47.9%, 293/612) had a missing RDT result, which was a consequence of the difficult logistics that had to be dealt with. Also, 104/357 (29.1%) RDT positives missed results of parasitological examination. This proportion was much higher than in Guinea in the same study, probably also due to the larger distances and limited transport options in rural DR Congo. This observation underlines the shortcomings of multi-step HAT diagnosis with lack of confirmation capacity in SSS. A number of potential HAT patients might not have reached parasitological examination in the CDT or might have gone to a CDT not included in this study. We suspect that the majority of these were probably false RDT positives or stage 1 patients with relatively non-severe symptomatology, as it is assumed that for individuals with neurological stage HAT symptoms, the probability for referral is higher as they feel sick. The loss of HAT patients along the diagnostic pathway during active screening was previously estimated to be almost 50% of the patients, who would not receive treatment as a result [27]. These numbers also demonstrate the importance and potential impact of a future “screen and treat” strategy for elimination of HAT [6]. Finally, only 1 out of the 3 RDTs evaluated in the DiTECT-HAT-WP2 study is still commercialised. The Abbott Bioline HAT 2.0, which is now commercialised and of which specificity, but not sensitivity, has recently been tested prospectively [10], was unavailable. |
|  |  | **27** | Implications for practice, including the intended use and clinical role of the index test | 19 | Line 358- 373: From a clinical point of view, the study results have several implications. The study allowed to confirm the excellent sensitivity of the HAT Sero K-SeT RDT, under field conditions, but highlights again the lack of specificity of the actual RDTs. The loss of RDT positive individuals, between serological screening and parasitological confirmation, underlines the potential of a “screen and treat” strategy with a safe and easy to use drug for elimination of HAT [6]. On the other hand, the suboptimal specificity of the HAT Sero K-SeT RDT might in a “screen and treat” strategy, result in overtreatment. Furthermore, the results confirm sub-optimal sensitivity of both trypanolysis and to a lesser extent, indirect ELISA/T.b. gambiense, underlining the risk of missing true HAT positives when applying only one of these tests, or applying them in serial. Our results also underline the need to improve molecular tests for remote testing. New storage systems with DNA/RNA stabilisation buffer and new molecular tests, including a Trypanozoon S2 RT-qPCR allow RNA and DNA conservation and detection, and are being evaluated further [6,10,28]. Despite the imperfections of the individual reference laboratory tests, parallel or serial combination of immunological and molecular test positivity, can be used to select either for maximum sensitivity and identify individuals at risk for HAT who merit to be followed-up further, or for maximum specificity, for epidemiological purposes. This principle is already applied as a remote test algorithm in the STROGHAT screen and treat study (NCT06356974) [6]. |
|  | **OTHER INFORMATION** |  |  |  |  |
|  |  | **28** | Registration number and name of registry | 20 | Line 380-5: Data Availability Statement  Metadata, the trial protocol and Standard Operating Procedures of all rapid diagnostic tests and reference laboratory tests are available under a CC-BY license via https://doi.org/10.23708/LTTOWL. The data that support the findings of this study have been deposited with restricted access in DataSuds repository (IRD, France) at https://doi.org/10.23708/LTTOWL. They cannot be publicly shared because of legal restriction. The access to the data is subject to approval and a data sharing agreement. |
|  |  | **29** | Where the full study protocol can be accessed | 20 | Line 380-5: Data Availability Statement  Metadata, the trial protocol and Standard Operating Procedures of all rapid diagnostic tests and reference laboratory tests are available under a CC-BY license via https://doi.org/10.23708/LTTOWL. The data that support the findings of this study have been deposited with restricted access in DataSuds repository (IRD, France) at https://doi.org/10.23708/LTTOWL. They cannot be publicly shared because of legal restriction. The access to the data is subject to approval and a data sharing agreement. |
|  |  | **30** | Sources of funding and other support; role of funders |  | Line 387-90: Funding  This study was funded by the EDCTP2 programme supported by the European Union (grant number DRIA-2014-306-DiTECT-HAT, VL). The funders had no role in study design, data collection and analysis, decision to publish, or preparation of the manuscript. |
|  |  |  |  |  |  |

.
