# Supplementary material for: Passive surveillance of human African trypanosomiasis in the Democratic Republic of the Congo: clinical presentation and prospective evaluation of rapid diagnostic and reference laboratory test accuracy
Source: PLoS Negl Trop Dis. 2025 Sep 29;19(9):e0013045. doi: 10.1371/journal.pntd.0013045 (PMC12503250; doi:10.1371/journal.pntd.0013045)
Supplement: Attachment — Submitted filename: response to reviewers.docx [file pntd.0013045.s003.docx]

# **Reviewers' comments:**

## 1. Methods

### 1.1 Reviewer #1:

**In the context of declining prevalence and the WHO's proposed SPTs, we feel that this study has clear objectives, and by using parasitology as the gold standard for confirming cases of HAT, the RDTs have been correctly evaluated.**

**The sample size could not be very large given the drop in prevalence, but selection on the basis of clinical suspicion is what is recommended.**

**To compensate for the small sample size, the multicentricity of the study provided sufficient power.**

**The statistical analyses used were sound, and ethical requirements were met.**

Authors Response: none required

### 1.2 Reviewer #2:

**The methods section describes accurately how the study was conducted, including information on the study protocol and the methods for data analysis. For the diagnostic assays, except for the RDTs, there is only a mention in the methods without detailed description whether they were produced in house or if they were supplied by a company. A more detailed description is required for the assays listed under “trypanolysis and indirect ELISA/T.b. gambiense and for Trypanozoon DNA by m18S qPCR and TgsGp-qPCR”.**

Authors Response: For all diagnostic assays (3 RDTs, trypanolysis, indirect ELISA/*T.b. gambiense*, *Trypanozoon* DNA by m18S qPCR and TgsGp-qPCR as well as LAMP) the standard operating procedures describing in detail the materials used and the manipulations carried out in a step by step way, are freely available at a public repository, which is opened by clicking on the provided link. The availability of these SOPS in the repository is mentioned several times in the manuscript:

- Methods section, study protocol: Line 153-155:”*The study documents and all standard operating procedures of tests mentioned below are deposited in* [*https://doi.org/10.23708/LTTOWL .*](%20https://doi.org/10.23708/LTTOWL%20.)”
- Methods section, reference laboratory tests: line 184: *“Standard Operation Procedures are available at* [*https://doi.org/10.23708/LTTOWL*](https://doi.org/10.23708/LTTOWL) *.”*
- Data availability statement: line 384: *“Metadata, the trial protocol and Standard Operating Procedures of all rapid diagnostic tests and reference laboratory tests are available under a CC-BY license via* [*https://doi.org/10.23708/LTTOWL*](https://doi.org/10.23708/LTTOWL) *.”*

We therefore invite the reviewer and the readers of the article, to click on this link and discover the 17 standard operating procedures available.

### 1.3 Reviewer #3:

**Under the data analysis section, the authors show that the specificity and sensitivity was analysed in graphpad, but its not clear at what level this was done. What was the gold standard for this and why and which test kit comparisons were done in comparison to the gold standard. More details need to be provided here. It is also not clear what specificity means in this case** **as suspected patients with negative parasitological outcome were excluded from the analysis, more information on what formed the specificity analysis needs to be provided.**

Authors Response: As suggested by the reviewer, we reorganised the paragraph on data analysis to improve clarity. We now first describe how the HAT status, which is the gold standard, was defined. Next, all formulas for calculating diagnostic performance, including diagnostic accuracy (see remark below) have been added. It is not clear to the authors what the reviewer means with “*as suspected patients with negative parasitological outcome were excluded from the analysis*” as, as shown in fig 2, all study participants with full RDT results and negative parasitological outcome (HAT negative population) have been taken into account. However, as shown in fig 2, 104 RDT positives needed to be excluded because no results for the parasitology gold standard were available (they did not present).

**The authors looked at clinical presentation and RDT testing in line with parasitilogical examination and molecular based testing. However, I do not see any relationship between the factors being studied. Why did the authors look at clinical presentation when they are testing diagnostic tests. My thinking was that they wanted to associate key clinical pictures with the diagnostic test outcomes to see if there is a clinical picture that could predict test outcome, eg do individuals who have a certain symptom/symptoms or a set of them more likely to be positive with a certain test and thus** **clinician can use this clinical picture to treat without parasitological confirmation. I would have loved to see this type of analysis. line 223 seems to be about this but on deeper reading it is not and instead (how this analysis for this section was done is not indicated in the data analysis section of the methods)**

Authors Response: In the methods section we have now better clarified why we evaluate the clinical presentation: *“Associations of clinical symptoms and signs with the HAT status were assessed by regression analysis (Statistical Package: SPSS) in order to identify, among the 13 clinical presentations used for inclusion in the study, which ones are most suited to better target RDT testing for HAT in passive screening.”* As correctly identified by the reviewer, the paragraph on *“Clinical presentation: association with HAT and diagnostic performance”* (initially line 223) is about this. As explained above, the analyses carried out have now been better defined in the methods section.

Furthermore, the role of the identified clinical signs in HAT diagnosis has been further underlined in the conclusion sections by adding the following sentence: “*Patients presenting with swollen lymph nodes, sleep disruption or psychiatric problems presenting at health facilities in HAT endemic zones, should undergo an RDT for HAT in DR Congo.*” (see also below)

## 2. Results

### 2.1 Reviewer #1:

**In reading the results, we note that the analyses made are based on the statements made in the plan; the various tables contribute to the understanding and synthesis of the quantitative results.
Data exclusions from the analysis have been justified.**

Authors Response: none required

### 2.2 Reviewer #2:

**The description of the results is clear and easy to follow. I just recommend the authors carefully check how the data is presented in the tables. For example, I could not figure out why in Table 1 there is a line named “Sex male” but no line for female.**

Authors Response: In the table, the gender was treated in the same way as the clinical presentation, so the number of males was given, the number of females can be easily deduced from that, and, in addition, is mentioned in the text: *“Most participants were female 1693/3113 (54.4%), …”*

### 2.3 Reviewer #3

**• I would have loved to see results on how the clinical picture relates to the diagnostic kit performance.**

Authors Response: The relation between the clinical picture and the HAT status, which serves as the gold standard throughout the whole manuscript, is described in the paragraph on *“Clinical presentation: association with HAT and diagnostic performance”.* In order to clarify that the HAT status was the gold standard for evaluation of the diagnostic performance of the clinical signs “*for HAT*” has been added in the sentences of this paragraph mentioning sensitivity and specificity.

**• The tables need to be improved for clarity in terms of the design and content. The column for the tests needs to show this and headings should be bolded for clarity**

Authors Response: Tables will be formatted by PLOS NTD after acceptation of the article. To simplify reviewers interpretation, column headings have been bolded.

**• Line 246. Am concerned about the 18.2% of the PPV of the HAT Sero K-SeT. If this is true then the authors need to check the analysis, otherwise it appears not to add up.**

Authors Response: The analysis was checked. The PPV was 42 HAT patients with positive HAT Sero *K*-Set over 231 HAT Sero *K*-Set positives in total, giving 18.2% PPV (42/231). The PPV is influenced by the disease prevalence (cfr. Reference Banoo 2010 which was added). The HAT prevalence among the study participants was 1.35%, this has now also been added in the descriptive analysis section.

**• Line 246, talks about diagnostic accuracy, I am not sure how this was calculated and needs to be indicated in the data analysis section of the paper.**

Authors Response: As requested, the calculation has now been added to the methods section, and the reference Banoo2010 (reference 20) was added: “*The sensitivity (number of test positives in the HAT test population, over the total HAT test population), specificity (number of test negatives in the non-HAT test population, over the total non-HAT test population), positive predictive value (PPV; number of test positives in the HAT test population, over the total number of test positives) and negative predictive value (NPV; number of test negatives in the non-HAT test population over the total number of test negatives) and diagnostic accuracy (number of test positives in the HAT test population plus number of test negatives in the non-HAT test population over the total number tested) for diagnosis of HAT were calculated [20], with 95% Wilson-Brown confidence intervals (Graphpad Prism 10.4.1)*.”

## 3. Conclusions

### 3.1 Reviewer #1:

**Yes, the conclusions are in line with the data presented, while noting the weaknesses due to the sample size, and the sensitivity and specificity of the various tests used.
The authors have shown that these RDTs, although having limitations, can be used especially in the test-and-treat strategy with non-toxic drugs and may contribute to the elimination of HAT, and they have recommended continuing studies with these RDTs in combination to increase sensitivity and to have a large sample size for subsequent evaluations.**

Authors Response: none required

### 3.2 Reviewer #2

**The findings as very relevant concerning both the epidemiology aspects and also the assessment of the performance of the diagnostic tests. A significant number of individuals was tested providing an indication on the level of HAT prevalence in the regions comprised in the study. In addition, the paper raises questions about the usefulness of the molecular tests for remote testing since their performance was very poor. The authors try to be positive, concluding that by combining different tests more conclusive data can be generated. However, they can emphasize in a stronger way that better tests are urgently needed.**

Authors Response: We thank the reviewer for this suggestion and added a last sentence emphasizing the need for better tests. Reference 30, corresponding to the TPP for a reference laboratory test, was also included: “*However, overall, better diagnostics, compliant to the respective target product profiles established by WHO [5,30], remain required to support elimination of HAT.*”

### 3.3 Reviewer #3

**The authors have tried to discuss the limitation of their study and this seems to make the discussion strong. The conclusions on molecular tests need to be improved and evaluated appears far fetched as the study is looking at clinical presentations and rapid diagnostic tests, for which no clear conclusion has been provided to this effect, see line 46.**

Authors Response: As suggested by the reviewer, a sentence on clinical presentation has been added to the concluding paragraph of the manuscript: *“**First, patients presenting with swollen lymph nodes, sleep disruption or psychiatric problems presenting at health facilities in HAT endemic zones in DR Congo, should undergo an RDT to screen for HAT.”*

The weak diagnostic performance, in particular of the molecular tests, cannot be ignored in the concluding section, as evaluating the diagnostic performance of reference laboratory tests was part of the objectives of the study, as mentioned in the last sentence of the introduction.

## 4. Editorial and Data Presentation Modifications?

### 4.1 Reviewer #1:

**No suggestions**

### 4.2 Reviewer #2:

**A request for additional information required in the methods section and a review of the tables in the results section are mentioned above.**

Authors Response: These adaptations have been made.

### 4.3 Reviewer #3:

**The authors need to improve the data analysis section of the methods to indicate the different tests for which results are being reported in the result section. the authors report in the results a relationship between the clinical picture and diagnostic outcome but this is not indicated in the analysis plan. i would also have like to see a section relating clinical picture of a set of clinical symptoms to diagnostic kit performance.**

Authors Response: The data analysis section was improved to clearly include evaluation of diagnostic performance of clinicals signs, RDTs and laboratory reference tests with HAT status as gold standard. We prefer not to add a section on the relationship between clinical data and indirect diagnostic test performance (RDTs and molecular test) as this was not an objective of the study.

## 5. Summary and General Comments

**Reviewer #1: This publication shows the efforts that partners must make to respond to the WHO's TPP in order to find tests that are easy to use in the context of falling prevalence, and above all what must be used to prove the cessation of transmission. There is still a great deal to be done, and researchers are encouraged to adapt even the methodologies to a particular context.**

**Reviewer #2: The paper describes the efforts to monitor human African trypanosomiasis (HAT) in several locations in the Democratic Republic of the Congo in the context of the WHO-driven effort to eliminate gambiense HAT. Over three thousand people have been tested over a three-year period between October 2017 and December 2020. The paper deals also with the limitations of rapid diagnostic test for monitoring HAT at screening centers and with the low efficiency of molecular tests for remote testing. Although rapid diagnostic tests also need improvement, the extremely low performance of the molecular tests is very concerning. All this information is relevant to be published.**

**Reviewer #3: The authors describe the clinical presentation and performance of selected RDTs and reference tests on gHAT diagnosis. The study offers promise on the diagnosis of HAT, although some of the kits might not be readily available. Although the study has limited novelty, the power of the study makes it interesting and provides insights how diagnostic kit performance. the weakness relates to the data analysis as described above and this could be improved by the research team.**

Authors Response to the 3 reviewers: Authors would like to thank the reviewers for their input. We have adapted the manuscript according to their suggestions.

## 6. Editorial Comments

**1) Please upload figure 1 as a separate Figure file in .tif or .eps format. For more information about how to convert and format your figure files please see our guidelines:** 
[**https://journals.plos.org/plosntds/s/figures**](https://journals.plos.org/plosntds/s/figures)

**Figure resubmission: While revising your submission, please upload your figure files to the Preflight Analysis and Conversion Engine (PACE) digital diagnostic tool,** [**https://pacev2.apexcovantage.com/**](https://pacev2.apexcovantage.com/)**. PACE helps ensure that figures meet PLOS requirements. To use PACE, you must first register as a user. Registration is free. Then, login and navigate to the UPLOAD tab, where you will find detailed instructions on how to use the tool. If you encounter any issues or have any questions when using PACE, please email PLOS at** [**figures@plos.org**](mailto:figures@plos.org)**. Please note that Supporting Information files do not need this step. If there are other versions of figure files still present in your submission file inventory at resubmission, please replace them with the PACE-processed versions.**

Authors Response: The figure has been uploaded in PACE and is resubmitted as a tif.

**2) We have noticed that you have uploaded Supporting Information files, but you have not included a list of legends. Please add a full list of legends for your** **Supporting Information files after the references list.**

Authors Response: Legends have been added as instructed.

**3) Some material included in your submission may be copyrighted. According to PLOSu2019s copyright policy, authors who use figures or other material (e.g., graphics, clipart, maps) from another author or copyright holder must demonstrate or obtain permission to publish this material under the Creative Commons Attribution 4.0 International (CC BY 4.0) License used by PLOS journals. Please closely review the details of PLOSu2019s copyright requirements here: PLOS Licenses and Copyright. If you need to request permissions from a copyright holder, you may use PLOS's Copyright Content Permission form.**

**Please respond directly to this email and provide any known details concerning your material's license terms and permissions required for reuse, even if you have not yet obtained copyright permissions or are unsure of your material's copyright compatibility. Once you have responded and addressed all other outstanding technical requirements, you may resubmit your manuscript within Editorial Manager. Potential Copyright Issues:**

**i) Figure 1. Please (a) provide a direct link to the base layer of the map (i.e., the country or region border shape) and ensure this is also included in the figure legend; and (b) provide a link to the terms of use / license information for the base layer image or shapefile. We cannot publish proprietary or copyrighted maps (e.g. Google Maps, Mapquest) and the terms of use for your map base layer must be compatible with our CC BY 4.0 license.**

**Note: if you created the map in a software program like R or ArcGIS, please locate and indicate the source of the basemap shapefile onto which data has been plotted.**

**If your map was obtained from a copyrighted source please amend the figure so that the base map used is from an openly available source. Alternatively, please provide explicit written permission from the copyright holder granting you the right to publish the material under our CC BY 4.0 license.**

**If you are unsure whether you can use a map or not, please do reach out and we will be able to help you. The following websites are good examples of where you can source open access or public domain maps:**

*** U.S. Geological Survey (USGS) - All maps are in the public domain. (**[**http://www.usgs.gov**](http://www.usgs.gov)**)**

*** PlaniGlobe - All maps are published under a Creative Commons license so please cite u201cPlaniGlobe,** [**http://www.planiglobe.com**](http://www.planiglobe.com)**, CC BY 2.0u201d in the image credit after the caption. (**[**http://www.planiglobe.com/?lang=enl**](http://www.planiglobe.com/?lang=enl)**)**

*** Natural Earth - All maps are public domain. (**[**http://www.naturalearthdata.com/about/terms-of-use/**](http://www.naturalearthdata.com/about/terms-of-use/)**).**

Authors Response: The figure has been adapted to be compliant with the PLOSu2019s copyright requirements

**Reproducibility: To enhance the reproducibility of your results, we recommend that authors of applicable studies deposit laboratory protocols in protocols.io, where a protocol can be assigned its own identifier (DOI) such that it can be cited independently in the future. Additionally, PLOS ONE offers an option to publish peer-reviewed clinical study protocols. Read more information on sharing protocols at** [**https://plos.org/protocols?utm_medium=editorial-email&utm_source=authorletters&utm_campaign=protocols**](https://plos.org/protocols?utm_medium=editorial-email&utm_source=authorletters&utm_campaign=protocols)

Authors Response: As mentioned under point 1.2 above and in the manuscript, the standard operating procedures (together with all study documents and data) are already available through a public data repository: <https://doi.org/10.23708/LTTOWL>
