# Supplementary material for: Passive surveillance of human African trypanosomiasis in the Democratic Republic of the Congo: clinical presentation and prospective evaluation of rapid diagnostic and reference laboratory test accuracy
Source: PLoS Negl Trop Dis. 2025 Sep 29;19(9):e0013045. doi: 10.1371/journal.pntd.0013045 (PMC12503250; doi:10.1371/journal.pntd.0013045)
Supplement: Attachment — Submitted filename: Rebutal R2bis.docx [file pntd.0013045.s004.docx]

Dear Editor,

Please find herewith our 2^nd^ revision. As shown below, we fully followed the additional editor comments adding a point of overestimating sensitivity to the limitations of the study.

We hope that these changes will lead to a swift acceptation of the manuscript.

Furthermore, we would like to underline that the data underlying the study, are not open because of legal restrictions, but can be obtained after contacting the address provided. In addition, we removed the text from figure 1.

Sincerely yours,

Veerle Lejon, corresponding author

**Additional Editor Comments:**

**“We would like to accept your manuscript for publication in PLoS NTDs but one issue came up that still needs to be resolved. There are concerns about the methodology, the way the samples have been selected. Ideally all samples should have been tested with a gold standard test, which in this case would be parasitology. But we understand that HAT is a rare disease, that parasitological confirmation tests are cumbersome and time consuming, and that for those reasons it is not feasible to do such tests on all samples in a field based prospective study. However, when samples included are selected based on three screening tests that also (partially) rely on the same antigens, there is a serious risk for bias, in particular for overestimating sensitivity. We kindly request you to explicitly address this in the limitations.”**

The following sentences were added to the limitation section of the discussion: “*Due to the lack of parasitological examination of all RDT negatives, and the fact that the evaluated RDTs largely rely on the same VSG antigens (complemented with ISG65 for rHAT Sero-Strip), we cannot exclude a risk of overestimation of sensitivity. Carrying out parasitology on all RDT negative participants would have been unfeasible in this field based prospective study, due to the additional work load, but also because of the lack of confirmation capacity in the SSS which would have obliged all participants to go to the CDT, or to limit the study to CDTs only.*”
